# Identifying an Immune-Related Gene ST8SIA1 as a Novel Target in Patients With Clear-Cell Renal Cell Carcinoma

**DOI:** 10.3389/fphar.2022.901518

**Published:** 2022-07-07

**Authors:** Xu Hu, Yanfei Yang, Yaohui Wang, Shangqing Ren, Xiang Li

**Affiliations:** ^1^ Institute of Urology, Department of Urology, West China Hospital, West China Medical School, Sichuan University, Chengdu, China; ^2^ The Third Xiangya Hospital of Central South Hospital, Changsha, China; ^3^ Robot Minimally Invasive Center, Sichuan Provincial People’s Hospital, Chengdu, China

**Keywords:** clear-cell renal cell carcinoma, immune-related, ST8SIA1, prognosis, target

## Abstract

Clear-cell renal cell carcinoma (ccRCC) is one of the most common urological cancers. The tumor microenvironment plays an important role in tumor development. The present study was conducted to identify novel immune-related biomarkers. The differentially expressed genes were identified using the ESTIMATE algorithm base on GEO and TCGA databases. The Kaplan–Meier survival curve and univariate and multivariate analyses were performed. The association between ST8SIA1 and the immune system was explored. The gene set enrichment analysis (GSEA) and online databases were used for functional annotation. ST8SIA1 was identified as a potential prognostic gene. Elevated ST8SIA1 was observed in the tumor tissues compared with adjacent normal tissues and associated with higher T stage and advanced TNM stage (all *p* < 0.05). The mRNA and protein levels of ST8SIA1 in cancer tissues and cells are also upregulated. The Kaplan–Meier survival curve and univariate and multivariate analyses showed that higher expression of ST8SIA1 was associated with worse OS (all *p* < 0.05). ST8SIA1 expression levels were negatively correlated with tumor purity and positively associated with infiltrated immune cells and expression of immune checkpoint genes. Function analysis also revealed that ST8SIA1 was significantly associated with immune-related pathways. In conclusion, ST8SIA1 was identified as an immune-related gene and a potential target in ccRCC patients. Further relevant studies are required to validate our findings.

## Introduction

Renal cancer is one of the most common urological cancers, with an estimated 431,288 new cases and 179,368 deaths in 2020 worldwide (Sung et al., 2021). Renal cell carcinoma (RCC) accounts for approximately 90% of all kidney malignancies (Ljungberg et al., 2019). RCC includes three main histological types: clear-cell RCC (ccRCC), papillary RCC, and chromophobe RCC; ccRCC is the most common (80–90%) and aggressive type (Ljungberg et al., 2019). For localized disease, surgical resection with curative intent is the standard treatment. However, approximately 20–30% of patients will develop local or distant recurrence after surgery (Williamson et al., 2016; Jamil et al., 2020). Moreover, about 30% of patients had metastatic diseases at initial diagnosis (Siegel et al., 2022). To be noted, the clinical outcome of advanced diseases is very poor and the 5-year relative survival rate for the distant-stage disease is only about 14% (Siegel et al., 2022).

Immunotherapy such as immune checkpoint inhibitors is becoming a promising treatment for advanced RCC (Bedke et al., 2021). Immune checkpoint inhibitors have shown encouraging results, which could improve outcomes of advanced or metastatic RCC (Motzer et al., 2019; Rini et al., 2019; Motzer et al., 2021). However, the response rates of patients who receive immune checkpoint inhibitors are not high. Reportedly, the tumor microenvironment plays an important role in the tumor development and response to immunotherapies (Binnewies et al., 2018). Moreover, ccRCC is also a highly immune cell-infiltrated cancer (Chevrier et al., 2017). Therefore, it is necessary to explore the potential prognostic genes in ccRCC patients that are immune-related.

The cells within the tumor microenvironment are an important component of the tumor tissue and strongly affect the behavior and malignancy of the tumor (Binnewies et al., 2018). Infiltrating immune and stromal cells are necessary components for the function of the tumor microenvironment, which are reported to be associated with tumor growth, recurrence, and metastasis (Hanahan and Coussens, 2012; Becht et al., 2016). An algorithm named Estimation of Stromal and Immune cells in Malignant Tumours using Expression Data (ESTIMATE) was designed to estimate the immune and stromal cells in malignant tumor tissues, which could also calculate the immune and stromal score (Yoshihara et al., 2013). Therefore, the present study aimed to explore the novel immune-related genes as prognostic factors in ccRCC patients based on The Cancer Genome Atlas (TCGA) and Gene Expression Omnibus (GEO) databases by applying the ESTIMATE algorithm.

## Materials and Methods

### Data Collection

The expression profiling data of GSE126964 (*n* = 66) were downloaded from the GEO database (http://www.ncbi.nlm.nih.gov/geo) to identify differentially expressed genes (DEGs). The RNA-seq and clinical data of ccRCC (TCGA-KIRC data) obtained from the TCGA data portal were downloaded from the University of California Santa Cruz (UCSC) Xena database (https://xenabrowser.net/datapages/). After excluding incomplete data, a total of 527 TCGA-KIRC patients were included in the analysis.

### Identification of Differentially Expressed Genes

The GSE126964 and TCGA-KIRC cohorts were divided into low and high score groups separately according to the median value of immune and stromal scores. DEGs were screened by using the package limma (version R 3.6.3). The criteria of DEGs selection are |log2 fold change (FC)|>1 and adjusted *p* < 0.05. DEGs selected from GSE126964 and TCGA-KIRC patients with low scores compared to those with high scores were shown via heatmap (Supplementary Figure S1). Furthermore, Venn diagrams (https://bioinfogp.cnb.csic.es/tools/venny/) were conducted to identify common DEGs (Supplementary Figure S1). The 12 common DEGs in GSE126964 and TCGA-KIRC were identified, including ST8SIA1. Gangliosides are important in tumorigenesis and the development of cancers (Liu et al., 2018). Biswas et al. also observed that select gangliosides (GM2, GD2, and GD3) were associated with T-cell dysfunction in RCC patients (Biswas et al., 2009). ST8SIA1, also known as GD3 synthase (GD3S), is highly expressed in several tumors and plays an important role in the development and progression of cancer. However, the exact mechanism of GD3S in RCC remains unknown. So, the ST8SIA1 was chosen for further analysis and identified as a potential key immune-related gene in the ccRCC patients.

### Survival and Statistical Analysis in the TCGA-KIRC Cohort

The patients were divided into low and high score groups based on the optimal cutoff value of gene expression, which is obtained using package survivalROC (version R 3.6.3). First, the expression level of the prognostic gene between tumor and normal tissues was compared. Furthermore, the pathological characteristics’ (pT, pN, pM, and stage) boxplots were also conducted based on TCGA-KIRC data by using the Wilcoxon signed-rank test. Overall survival (OS) was compared between the low and high score groups by applying the Kaplan–Meier survival curve and the log-rank test. Univariate and multivariate analyses were also conducted to identify the prognostic factors of OS. In addition, the nomogram of OS was constructed based on the multivariate analysis. Furthermore, the concordance index (C-index), the calibration curve, and the decision curve analysis (DCA) were generated to evaluate the performance of the nomogram. All statistical analyses were carried out using R software version 3.6.3. Additionally, the comparison of mRNA expression levels of ST8SIA1 between ccRCC and normal tissue was also validated based on the Oncomine database (https://www.oncomine.org/) (Rhodes et al., 2004). The cBioPortal (https://www.cbioportal.org/) database was applied to acquire genomic alteration of ST8SIA1 in KIRC from TCGA (Cerami et al., 2012).

### The Association Between Prognostic Gene and Immune Microenvironment

TISIDB (http://cis.hku.hk/TISIDB/index.php) was used to investigate correlations between tumors and the immune system (Ru et al., 2019). To explore the correlation of the expression level of ST8SIA1 with immune cell infiltration level and expression of immune checkpoints genes, TIMER 2.0 (http://timer.cistrome.org/) was used (Li et al., 2020). In addition, the expression correlation of ST8SIA1 with immune checkpoints genes was also evaluated using the GEPIA ((http://gepia.cancer-pku.cn/) database (Tang et al., 2017). There were significant correlations when the *p*-value <0.05 and |Rho|>0.1.

### The Functions of Prognostic Gene in TCGA-KIRC

The gene set enrichment analysis (GSEA) 4.0.2 (https://www.gsea-msigdb.org/gsea/index.jsp) was applied to explore the relationship between the ST8SIA1 and HALLMARK pathways. ST8SIA1 was divided into low and high score categories to annotate phenotype. Based on the default values of parameters, the 1,000 random sample permutations were conducted. LinkedOmics (http://www.linkedomics.org/login.php), a publicly available portal including multi-omics data from TCGA, was used to obtain the genes associated with ST8SIA1 in KIRC and relevant functions, including the KEGG pathway and Gene Ontology (GO) (Vasaikar et al., 2018). CancerSEA (http://biocc.hrbmu.edu.cn/CancerSEA/home.jsp), the database that uncovers functional states of cancer cells at a single-cell resolution, was applied to explore the function of ST8SIA1 at a single-cell resolution (Yuan et al., 2019).

### Quantitative Real-Time Polymerase Chain Reaction

The HK-2, 786-O, and ACHN cell lines were obtained from American Type Culture Collection (ATCC). Total RNA from cells was isolated using a RaPure Total RNA Kit (Magen Biotechnology, China) according to the manufacturer’s manual. Complementary DNA (cDNA) was synthesized by using oligo (dT) primer and the RevertAid First Strand cDNA Synthesis Kit (Thermo Fisher Scientific, United States). Quantitative real-time PCR was performed using the QuantiNova SYBR Green PCR Kit (Qiagen, Germany) in a PCR system (Bio-Rad, United States). The PCR conditions were as follows: initial denaturation at 95°C for 2 min, followed by 95°C for 5 s and 60°C for 10 s for cycles. The gene primers were as follows: ST8SIA1, forward 5’-TAC​TCT​CTC​TTC​CCA​CAG​G-3’, and reverse 5’-GAC​AAA​GGA​GGG​AGA​TTG​C-3’. Relative gene expression was calculated using the 2^−ΔΔCt^ method and normalized with GAPDH.

### Immunohistochemical Analysis

The 46 ccRCC tumor tissues and 37 adjacent normal kidney tissues were used for immunohistochemistry. Then, xylene and a graded alcohol series were used for deparaffinization and hydration. After the citric acid solution was applied for antigen retrieval, the sections were incubated with normal goat serum for 30 min and primary antibody against ST8SIA1 (1:200; 24918-1-AP; ProteinTech Group, Inc.) at 4 °C overnight. Subsequently, the sections were incubated with secondary antibodies at room temperature for 30 min, and staining was performed using DAB. Then, the sections were counterstained with hematoxylin and observed under a microscope.

The immunohistochemical score was evaluated using the criterion reported previously (Zhu et al., 2020). The staining intensity level ranged from 0 to 3 (no staining, weakly positive, moderately positive, and strongly positive). Based on the fraction of positively stained tumor cells, the score ranged from 0 to 4 (negative, ≤ 25%, 26–50%, 51–75%, and > 75%). The final score was calculated by multiplying these two scores, ranging from 0 to 12 (−; +; ++; +++), and ≤3 was defined as low expression.

### Statistical Analysis

All statistical analyses were performed by an online database or R software. Two-sided *p*-values less than 0.05 were considered statistically significant.

## Results

### The Identification of DEGs in ccRCC Patients

The gene expression profiles were compared between low and high score groups based on the immune/stromal scores in the GSE126964 and TCGA-KIRC cohorts. In the GSE126964 dataset, 201 DEGs (2 upregulated and 199 downregulated genes) and 391 DEGs (24 upregulated and 367 downregulated genes) were obtained based on the differences in stromal and immune scores, respectively. Similarly, in the TCGA-KIRC cohort, the comparisons based on the stromal and immune scores generated 572 DEGs (20 upregulated and 552 downregulated genes) and 669 DEGs (18 upregulated and 651 downregulated genes), respectively. As shown in the Venn diagrams, only 12 DEGs were commonly downregulated both in the GSE126964 and TCGA-KIRC cohorts (Supplementary Figure S1). The ST8SIA1 (ST8 alpha-N-acetyl-neuraminide alpha-2,8-sialyltransferase 1) was identified as a potential key immune-related gene in the ccRCC patients.

### Association Between ST8SIA1 Expression and Clinicopathological Characteristics

**FIGURE 1:**
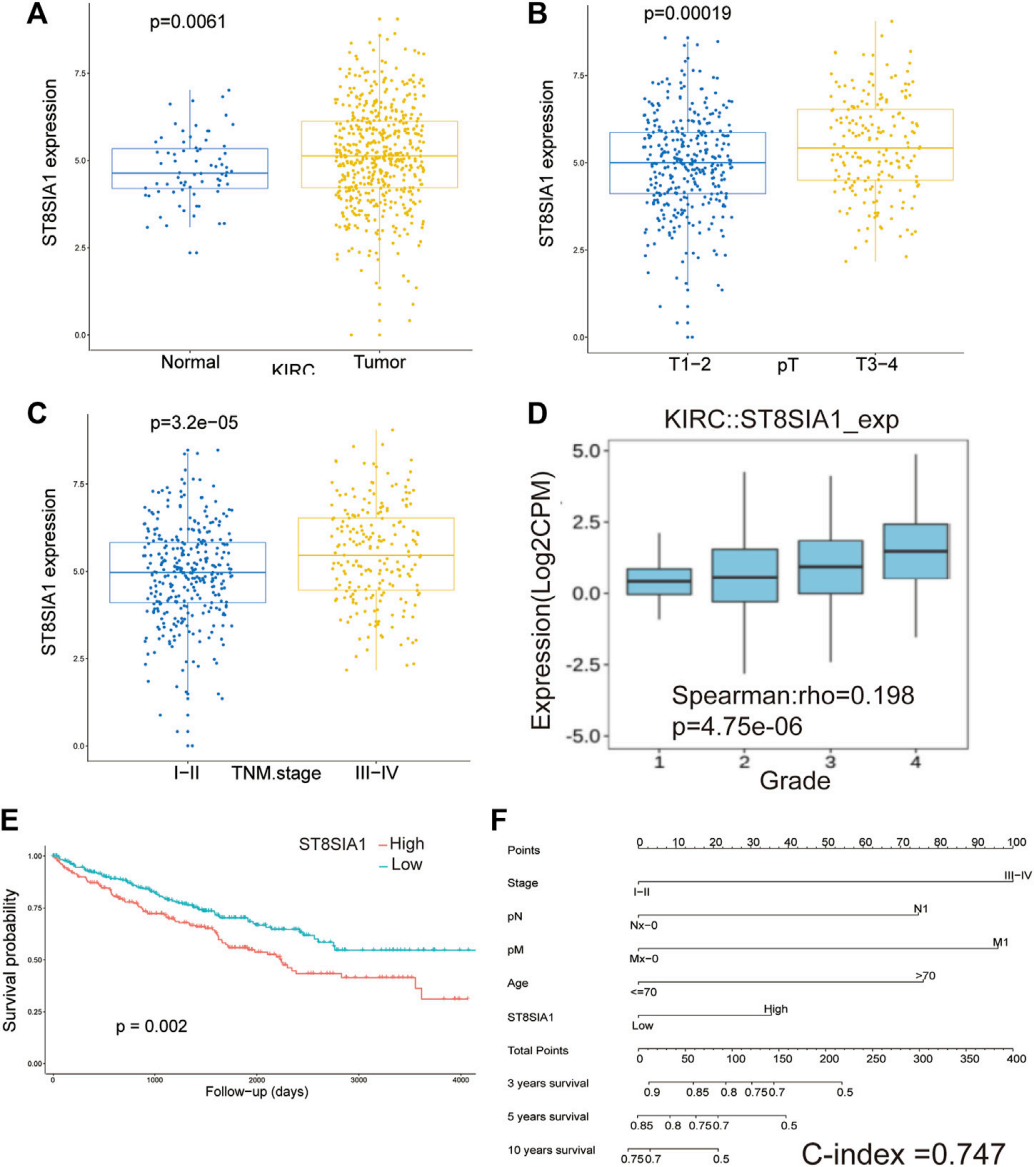
The mRNA expression level of ST8SIA1 in renal cancer and adjacent normal tissues in TCGA-KIRC **(A)**; the mRNA expression level of ST8SIA1 between the different T stage **(B)** and TNM stage **(C)** in TCGA-KIRC; the correlation between ST8SIA1 mRNA expression and tumor grade based on the TISIDB database **(D)**; the Kaplan–Meier curve analysis of OS grouped by ST8SIA1 expression (using optimal cutoff value) **(E)**; nomogram incorporating ST8SIA1 expression predicts probability of OS **(F)**.

The expression of ST8SIA1 was higher in the KIRC tissue compared with adjacent normal tissue (*p* = 0.006; Figure 1A). In the Oncomine database, meta-analysis also revealed ST8SIA1 was highly expressed in the tumor tissue (*p* = 0.03; Supplementary Figure S2). In the TCGA-KIRC cohort, higher expression of ST8SIA1 was significantly associated with advanced T stage and TNM stage (all *p* < 0.001; Figures 1B,C). Furthermore, high ST8SIA1 expression may be associated with lymph node metastasis and distant metastasis but did not reach a significant difference (Supplementary Figure S2). ST8SIA1 expression was also positively correlated with the grade of tumor (Rho = 0.198, *p* < 0.001; Figure 1D). The Kaplan–Meier curve revealed that higher ST8SIA1 expression (using optimal cutoff value) was associated with worse OS (*p* = 0.002; Figure 1E). The univariate analysis demonstrated that age, T stage, N stage, M stage, TNM stage, and ST8SIA1 expression were associated with OS (Table 1). Moreover, the multivariate analysis also revealed that higher ST8SIA1 expression was significantly associated with worse OS (*p* = 0.015; Table 1). Based on the multivariate analysis, the nomogram of OS was constructed (Figure 1F), with a C-index of 0.747. The calibration plots for predicting OS fitted well between the nomogram-predicted probability and actual observation at 3- and 5-year follow-ups (Supplementary Figure S2). Based on the DCA curves (Supplementary Figure S2), the nomogram showed larger net benefits across a wide range of threshold probability than the AJCC stage model both for 3-year and 5-year OS, indicating better clinical utilities. As for genomic alteration, ST8SIA1 was rarely mutated with a relatively low frequency of 1.1% based on the cBioportal database, indicating ST8SIA1 is highly conserved (Supplementary Figure S3).

**TABLE 1:** Univariate and multivariate Cox regression analyses of overall survival for clear-cell renal cell carcinoma patients in TCGA cohort.

Variables	Univariate analysis			Multivariate analysis		
	HR	95% CI	*p*-value	HR	95% CI	*p*-value
Age (>70 vs. ≤ 70)	1.84	1.35–2.52	<0.001^a^	2.37	1.72–3.21	<0.001^a^
Gender (female vs. male)	0.93	0.68–1.26	0.631			
Year of diagnosis (>2006 vs. ≤ 2006)	0.83	0.57–1.2	0.323			
T stage (T3–4 vs. T1–2)	3.12	2.31–4.23	<0.001^a^	1.03	0.56–1.88	0.929
N stage (N1 vs. NX-0)	3.85	2.13–7.14	<0.001^a^	2.13	1.12–4.17	0.021^a^
M stage (M1 vs. MX-0)	4.35	3.23–5.88	<0.001^a^	2.78	1.89–4	<0.001^a^
TNM stage (Ⅲ–Ⅳ vs. Ⅰ–Ⅱ)	3.82	2.79–5.24	<0.001^a^	2.30	1.15–4.59	0.019^a^
ST8SIA1 expression (high vs. low)	1.59	1.18–2.13	0.002^a^	1.45	1.08–1.96	0.015^a^

a *p*<0.05.

### Association Between ST8SIA1 Expression and Immune Cell Infiltration Level

**FIGURE 2:**
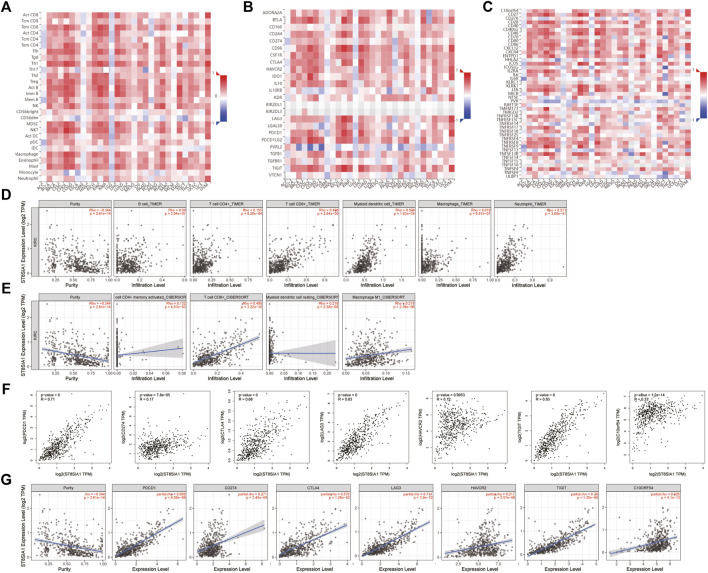
correlation analysis of ST8SIA1 expression and abundance of tumor-infiltrating lymphocytes **(A)**, immunoinhibitory **(B)**, and immunostimulatory **(C)** genes expression across different cancers from TCGA using the TISIDB database, KICH: chromophobe renal cell carcinoma, KIRC: clear renal cell carcinoma, KIRP: renal papillary cell carcinoma; The association of ST8SIA1 expression with immune cell infiltration levels in KIRC using the TIMER **(D)** and CIBERSORT **(E)** algorithms; The association of ST8SIA1 expression with immune checkpoint genes’ expression in KIRC using GEPIA **(F)** and TIMER 2.0 **(G)** databases.

Based on the TISIDB database, the correlation between the expression level of ST8SIA1 and the immune system was explored. As shown in Figures 2A–C, the ST8SIA1 expression level was positively correlated to the abundance of most tumor-infiltrating lymphocytes and immunoinhibitory and immunostimulatory gene expressions across different cancers from TCGA. ST8SIA1 was also found to be associated with immune subtypes in KIRC (Supplementary Figure S4), we found that ST8SIA1 expression was the highest in the C2 subtype (IFN-gamma dominant) and the lowest in the C5 subtype (immunologically quiet). Based on the TIMER and CIBERSORT algorithms, a negative relationship between ST8SIA1 expression and tumor purity was observed (Rho = -0.334; *p* < 0.001; Figures 2D,E). Conversely, the TIMER algorithm (Figure 2D) revealed that ST8SIA1 expression was positively associated with the infiltrating levels of CD4^+^ T cell (Rho = 0.155, *p* < 0.001), CD8^+^ T cell (Rho = 0.498, *p* < 0.001), neutrophils (Rho = 0.571, *p* < 0.001), and dendritic cells (Rho = 0.528, *p* < 0.001). The CIBERSORT algorithm (Figure 2E) demonstrated that the infiltration levels of M1 macrophage (Rho = 0.218, *p* < 0.001), activated CD4^+^ memory T cell (Rho = 0.132, *p* < 0.001), CD8^+^ T cell (Rho = 0.498, *p* < 0.001), and resting myeloid dendritic cell (Rho = 0.218, *p* < 0.001) were positively associated with ST8SIA1 expression. Furthermore, the arm-level gain of ST8SIA1 had a significant negative correlation with the infiltration level of CD8^+^ T cells in KIRC (Supplementary Figure S4).

### Association Between the Expression Levels of ST8SIA1 and Immune Checkpoint Genes

PDCD1 (PD-1), CD274 (PD-L1), and CTLA4 are well-known important immune checkpoint genes. Several novel immune checkpoints genes have been proposed recently, such as LAG3, HAVCR2 (TIM3), TIGIT, and VSIR (C10orf54) (Morad et al., 2021). Based on the GEPIA database (Figure 2F), the expression level of ST8SIA1 was significantly positively associated with the expression of PDCD1 (Rho = 0.71, *p* < 0.001), CD274 (Rho = 0.17, *p* < 0.001), CTLA4 (Rho = 0.68, *p* < 0.001), LAG3 (Rho = 0.83, *p* < 0.001), HAVCR2 (Rho = 0.12, *p* = 0.005), TIGIT (Rho = 0.83, *p* < 0.001), and VSIR (Rho = 0.33, *p* < 0.001). Adjusted by tumor purity (Figure 2G), the ST8SIA1 expression level was also significantly positively associated with the expression of PDCD1 (Rho = 0.699, *p* < 0.001), CD274 (Rho = 0.271, *p* < 0.001), CTLA4 (Rho = 0.579, *p* < 0.001), LAG3 (Rho = 0.711, *p* < 0.001), HAVCR2 (Rho = 0.217, *p* = 0.005), TIGIT (Rho = 0.79, *p* < 0.001), and VSIR (Rho = 0.329, *p* < 0.001).

### The Function of ST8SIA1 in KIRC

**FIGURE 3:**
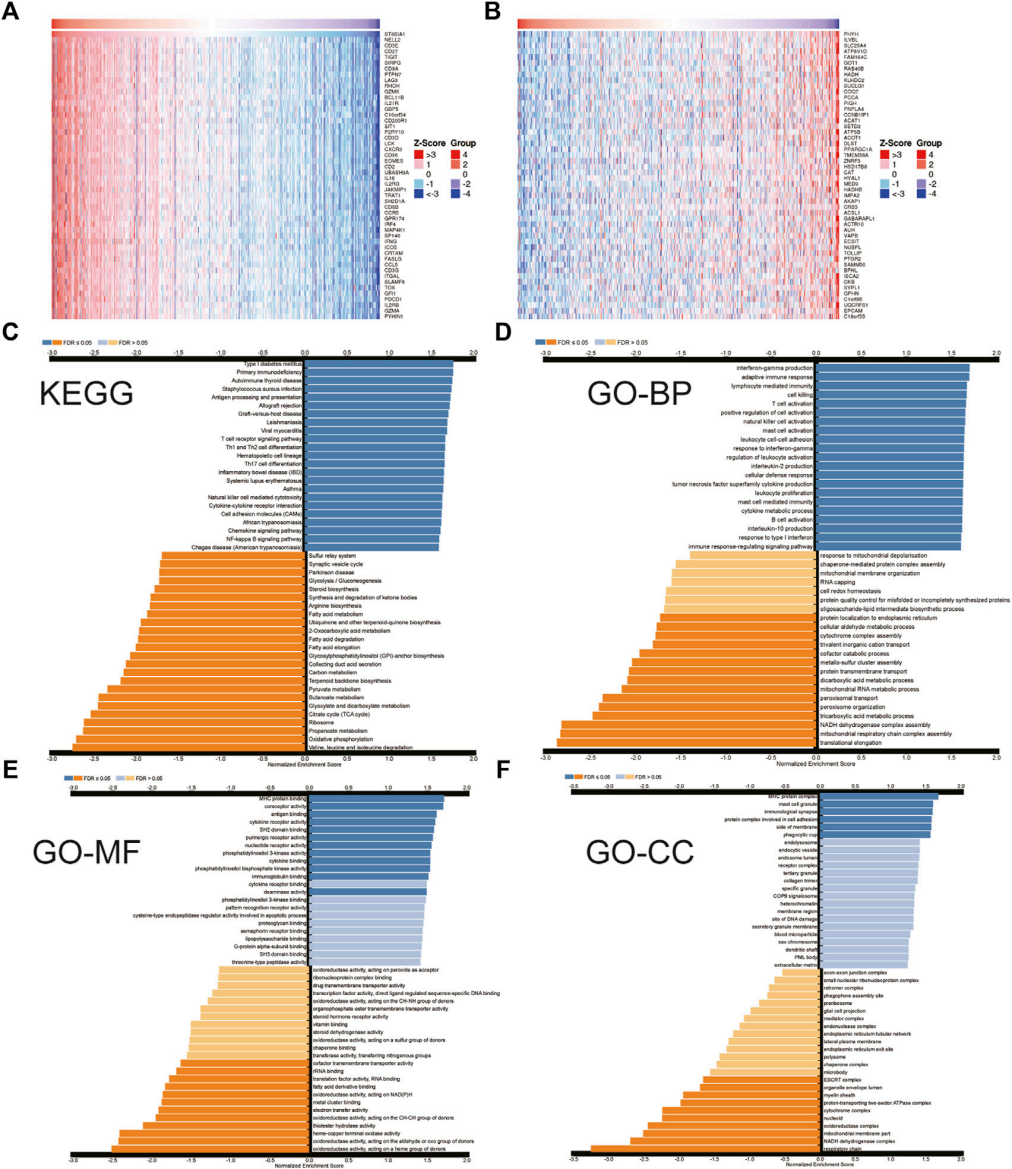
Heatmap of top 50 genes that have positive **(A)** and negative **(B)** correlations with ST8SIA1 expression in TCGA-KIRC using the LinkedOmics database; the KEGG pathway **(C)** and Gene Ontology (GO) biological process (BP), molecular function (MF), cellular component (CC) enrichment **(D–F)** of genes coexpressed with ST8SIA1.

The hallmark pathways were identified between low and high ST8SIA1 expression in the TCGA-KIRC cohort using GESA. Almost all of the top 10 pathways that correlated with elevated ST8SIA1 expression are immune-related (Table 2). Based on the LinkedOmics database, genes coexpressed with ST8SIA1 in KIRC were identified. The heatmaps show the top 50 genes, the expression of which exhibited significant positive or negative correlation with ST8SIA1 expression (Figures 3A,B). The GO and KEGG analyses of significant coexpressed genes showed that most enriched pathways are immune-related (Figures 3C–F). Furthermore, single-cell analysis using CancerSEA was performed to investigate the functions of ST8SIA1. In a single-cell resolution in RCC, ST8SIA1 expression was positively associated with the expression of stemness and differentiation signature genes (Supplementary Figure S4).

**TABLE 2:** The enrichment analysis of Hallmark pathway associated with ST8SIA1 expression using GSEA.

Name	NES	NOM p-val	FDR q-val
HALLMARK_ALLOGRAFT_REJECTION	2.737	0	0
HALLMARK_INTERFERON_GAMMA_RESPONSE	2.702	0	0
HALLMARK_INTERFERON_ALPHA_RESPONSE	2.642	0	0
HALLMARK_INFLAMMATORY_RESPONSE	2.328	0	0
HALLMARK_IL6_JAK_STAT3_SIGNALING	2.318	0	0
HALLMARK_EPITHELIAL_MESENCHYMAL_TRANSITION	2.155	0	0
HALLMARK_COMPLEMENT	2.142	0	0
HALLMARK_KRAS_SIGNALING_UP	1.983	0	0
HALLMARK_TNFA_SIGNALING_VIA_NFKB	1.956	0	0
HALLMARK_IL2_STAT5_SIGNALING	1.952	0	0

NES: normalized enrichment score; NOM: nominal; FDR: false discovery rate.

### The mRNA and Protein Level of ST8SIA1 in Tissue and Cell

**FIGURE 4:**
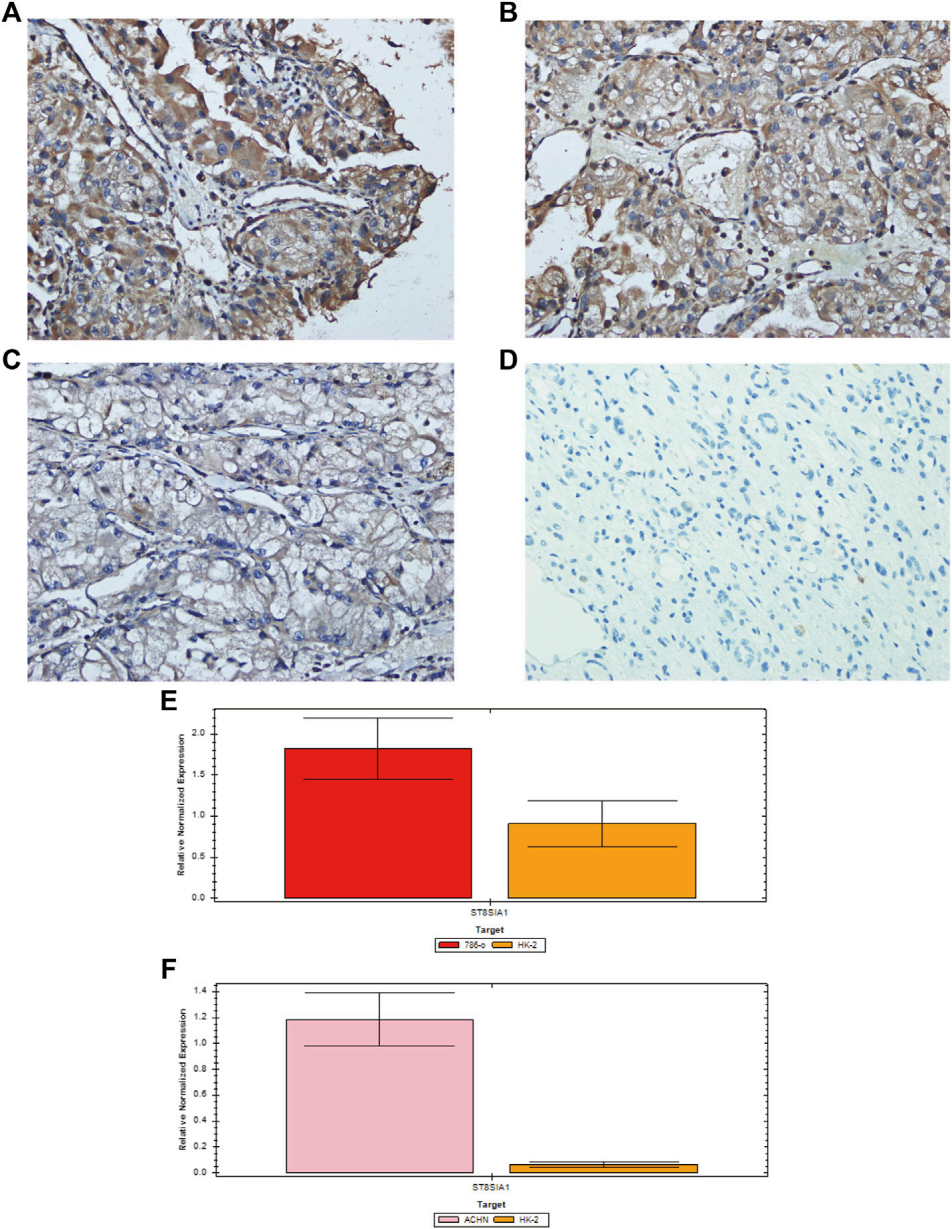
Representative immunohistochemical staining of ST8SIA1 protein in ccRCC tissues [**(A)** +++, **(B)** ++, **(C)** +, magnification: × 400] and normal kidney tissue [**(D)** −, magnification: × 400]; Quantitative real-time PCR of ST8SIA1 in 786-O **(E)** and ACHN **(F)** cells compared with HK-2 cell.

To verify the expression level of ST8SIA1, we performed the immunohistochemical analysis of 46 ccRCC tumor tissues and 37 adjacent normal kidney tissues (Figures 4A–D). We observed that 19 tumor tissues out of 46 have a >3 score, and 5 normal tissues of 37 have a >3 score (19/46 vs. 5/37; *p* < 0.05). A total of 8 tumor tissues out of 46 have a > 6 score, while no normal tissue has a > 6 score. To validate the selective expression of ST8SIA1 in tumor cells, the quantitative real-time PCR in RCC cell lines (786-O and ACHN) and a normal kidney epithelial cell line (HK-2) was performed. The ST8SIA1 mRNA was highly expressed in RCC cell lines, compared with a normal kidney cell line (Figures 4E,F).

## Discussion

The tumor microenvironment, which consists of tumor cells, and various infiltrating immune and stromal cells, plays a critical role in tumor growth, progression, and drug resistance (Binnewies et al., 2018). ccRCC is a highly vascularized and immune cell–infiltrated cancer, resulting in two revolutionary therapies including antiangiogenic therapy and immunotherapy (Qian et al., 2009; Chevrier et al., 2017). While a heterogeneous tumor microenvironment might be associated with therapy resistance and low response rate, it may affect the prognosis of patients.

With the wide application of bioinformatics, several studies have reported different immune-related genes that are associated with the prognosis of cancer patients. For example, Du et al. also revealed an immune-related prognostic factor (TGFBI) in ccRCC patients (Du et al., 2020). In addition, Liu et al. demonstrated that type 2 papillary RCC is associated with immune infiltration and explored potential new targets (CCL19/CCR7, CXCL12/CXCR4, and CCL20/CCR6) (Liu et al., 2020). In the present study, we screened immune-related DEGs based on the stromal/immune scores using the ESTIMATE algorithm in the GSE126964 and TCGA-KIRC cohorts. ST8SIA1 was identified as the immune-related prognostic gene after reviewing relevant research studies. We found that ST8SIA1 was more expressed in the KIRC tissue than adjacent normal tissue. Higher expression of ST8SIA1 was significantly associated with higher T stage and advanced TNM stage, which indicated that ST8SIA1 was associated with survival. Moreover, univariate and multivariate analyses also revealed that ST8SIA1 was associated with worse OS. Based on TIMER, TISIDB, and GEPIA databases, we observed that ST8SIA1 was significantly associated with infiltration levels of various immune cells. However, different immune cells play different roles in antitumor immunity. Furthermore, the ST8SIA1 expression was positively associated with the expression of several immune checkpoints, indicating a potential suppressive antitumor immunity. GSEA and the LinkedOmics database revealed that ST8SIA1 was significantly enriched in immune-related pathways and functions. All aforementioned findings suggested that ST8SIA1 was highly associated with the immune system and may be a potential target, but these results were obtained through bioinformatics; further studies are required to explore its exact role.

ST8SIA1, also known as GD3 synthase (GD3S), is highly expressed in several tumors, and plays an important role in the development and progression of cancer. In human melanoma SK-MEL-2 cells, GD3S is highly expressed and regulated by transcription factors NF-κB (Kang et al., 2007). Inhibition of GD3S could decrease the cell viability of melanoma cells (Kang et al., 2007). Ramos et al. have found that high expression of GD3S is associated with the phenotype of melanoma brain metastasis, and the overall survival is significantly worse. ST8SIA1 overexpression enhanced cell proliferation and colony formation in melanoma cells (Ramos et al., 2020). In human breast tumors, GD3S could lead to increased stem cell properties and metastatic competence via activation of the c-Met signaling pathway (Sarkar et al., 2015). Glioma is also a highly malignant tumor with a high recurrence rate. GD3S is also highly expressed in glioma. Suppression of GD3S could decrease glioma stem cell–associated properties (Yeh et al., 2016). Ko et al. found that GD3S was highly expressed in lung cancer, and inhibition of GD3S by siRNA could reduce the expression of GD2 and inhibit cell proliferation, migration, and invasion (Ko et al., 2006).

The enzyme GD3S is involved in the synthesis of disialogangliosides with three glycosyl groups (GD3) and is important in tumorigenesis and the development of cancers (Liu et al., 2018). Certain gangliosides, such as GD3, can promote tumor-associated angiogenesis and strongly regulate cell adhesion and thus initiate tumor metastasis. Moreover, ganglioside antigens on the cell surface, or shed from the cells, act as immunosuppressors (Birklé et al., 2003). In ovarian cancer, GD3 inhibits the NKT cell response as an immune escape mechanism via binding the CD1d antigenic-binding site (Webb et al., 2012). In cutaneous T-cell lymphoma, GD3 inhibits the production of IL-17A as a mechanism of suppressive antitumor immunity (Kume et al., 2021). Biswas et al. observed that selected gangliosides (GM2, GD2, and GD3) are associated with T-cell dysfunction in RCC patients (Biswas et al., 2009). However, the exact mechanism of GD3S in RCC remains unknown. Based on GSEA, we observed that interferon-γ, interferon-α, and IL6-JAK-STAT3 pathways were enriched, which may be connected with the upregulation of immune checkpoint genes and thus may play a role in suppressive antitumor immunity. Further experimental studies are required to explore the exact mechanism.

There are some advantages and limitations in the present study. GEO and TCGA have relatively large sample sizes and comprehensive genomic data, providing a good foundation for analysis. To our knowledge, there is no report that focuses on ST8SIA1 expression in ccRCC patients. Furthermore, we explored the function of ST8SIA1 and its association with immune systems. We also performed immunohistochemical analysis and quantitative real-time PCR to validate the expression level of ST8SIA1. However, though the present study was well-designed and performed carefully, the exact mechanism of ST8SIA1 in ccRCC patients still needs to be explored through relevant experiments.

In conclusion, higher expression of ST8SIA1 was associated with adverse factors as well as worse overall survival. ST8SIA1 was identified as an immune-related gene and potential target in ccRCC patients. Further relevant studies are required to validate our findings.

## Data Availability

The datasets presented in this study can be found in online repositories. The names of the repository/repositories and accession number(s) can be found in the article/Supplementary Material.
