# Supplementary figures and images for: Identifying an Immune-Related Gene ST8SIA1 as a Novel Target in Patients With Clear-Cell Renal Cell Carcinoma
Source: Front Pharmacol. 2022 Jul 7;13:901518. doi: 10.3389/fphar.2022.901518 (PMC9300832; doi:10.3389/fphar.2022.901518)

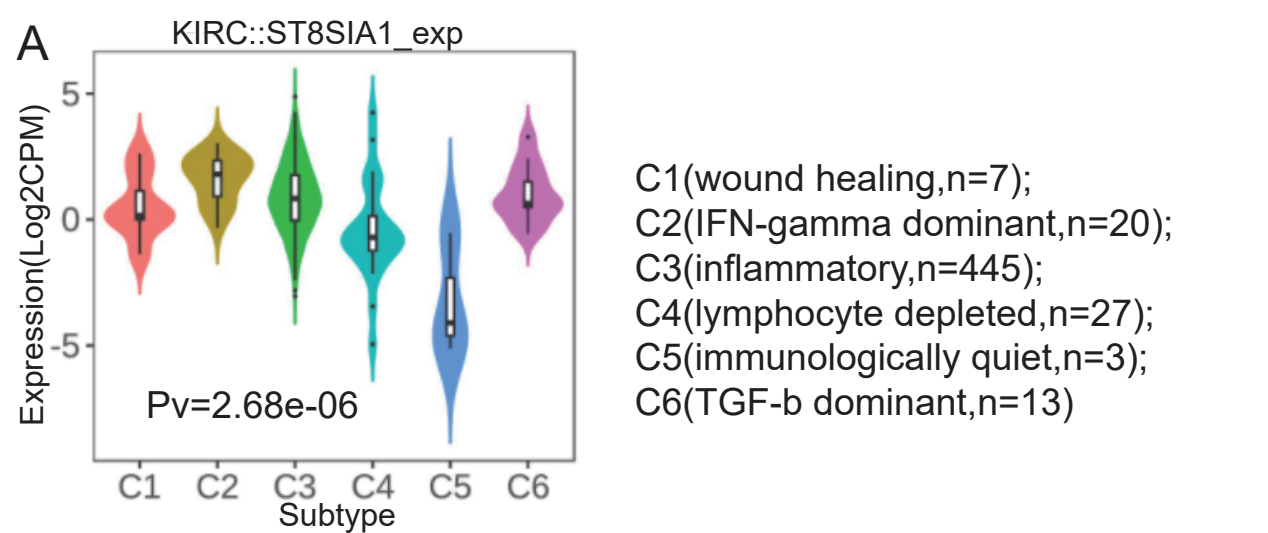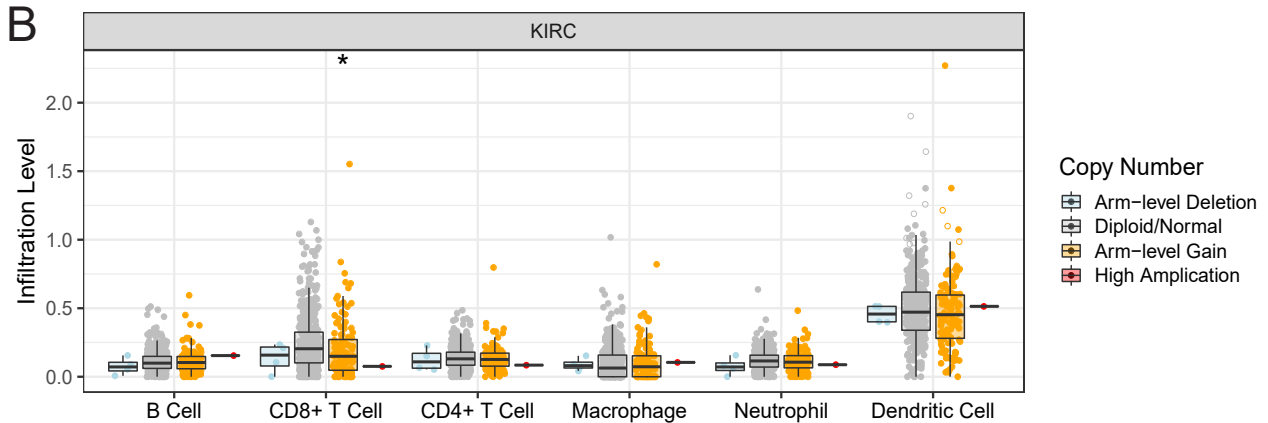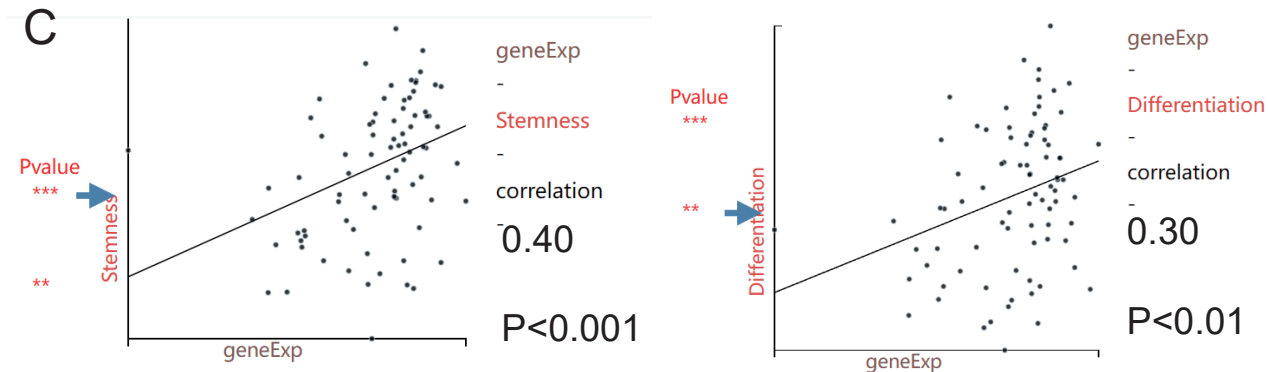

Supplement: Supplementary file 1 [file Image4.PDF]

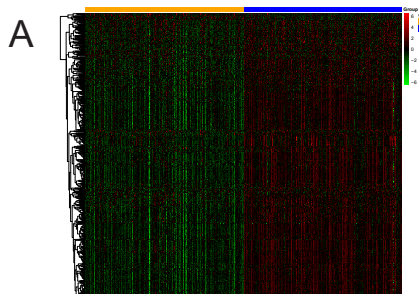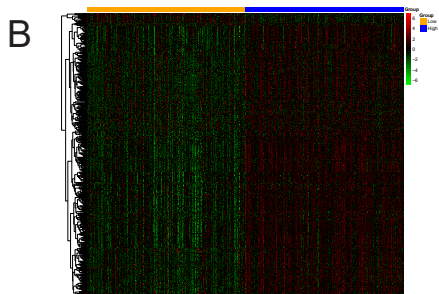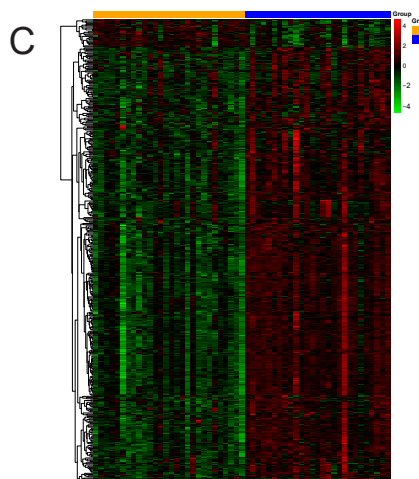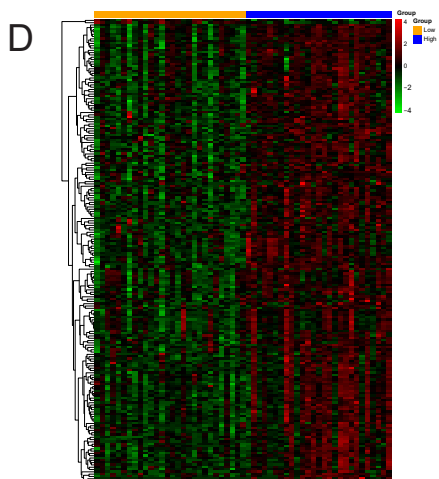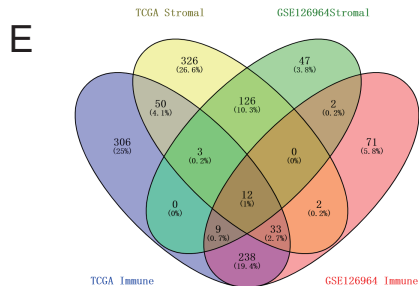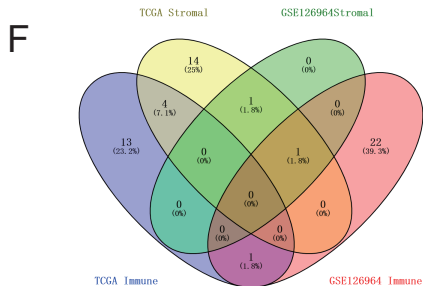

Supplement: Supplementary file 4 [file Image1.pdf]
