# Supplementary material for: Identifying an Immune-Related Gene ST8SIA1 as a Novel Target in Patients With Clear-Cell Renal Cell Carcinoma
Source: Front Pharmacol. 2022 Jul 7;13:901518. doi: 10.3389/fphar.2022.901518 (PMC9300832; doi:10.3389/fphar.2022.901518)
Supplement: Supplementary file 2 [file Image2.PDF]

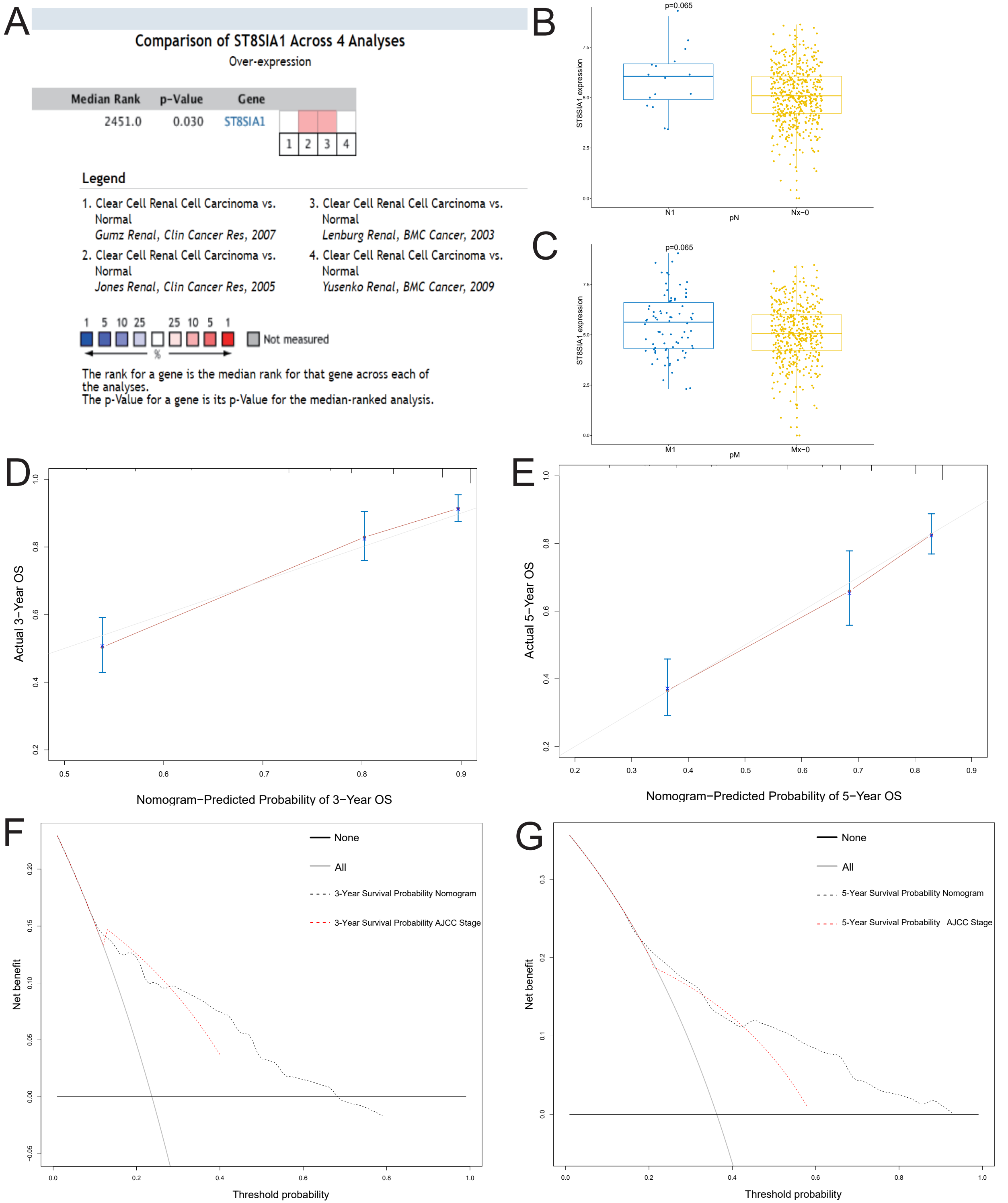

A
Comparison of ST8SIA1 Across 4 Analyses
Over-expression
Median Rank
p-Value
Gene
2451.0
0.030
ST8SIA1
1 2 3 4
Legend
1. Clear Cell Renal Cell Carcinoma vs. Normal
Gumz Renal, Clin Cancer Res, 2007
2. Clear Cell Renal Cell Carcinoma vs. Normal
Jones Renal, Clin Cancer Res, 2005
3. Clear Cell Renal Cell Carcinoma vs. Normal
Lenburg Renal, BMC Cancer, 2003
4. Clear Cell Renal Cell Carcinoma vs. Normal
Yusenko Renal, BMC Cancer, 2009
1 5 10 25 25 10 5 1
Not measured
%
The rank for a gene is the median rank for that gene across each of the analyses.
The p-Value for a gene is its p-Value for the median-ranked analysis.
B
p=0.065
ST8SIA1 expression
N1
Nx−0
pN
C
p=0.065
ST8SIA1 expression
M1
Mx−0
pM
D
Actual 3−Year OS
Nomogram−Predicted Probability of 3−Year OS
E
Actual 5−Year OS
Nomogram−Predicted Probability of 5−Year OS
F
None
All
3-Year Survival Probability Nomogram
3-Year Survival Probability AJCC Stage
Net benefit
Threshold probability
G
None
All
5-Year Survival Probability Nomogram
5-Year Survival Probability AJCC Stage
Net benefit
Threshold probability
