# Supplementary material for: Identifying an Immune-Related Gene ST8SIA1 as a Novel Target in Patients With Clear-Cell Renal Cell Carcinoma
Source: Front Pharmacol. 2022 Jul 7;13:901518. doi: 10.3389/fphar.2022.901518 (PMC9300832; doi:10.3389/fphar.2022.901518)
Supplement: Supplementary file 3 [file Image3.pdf]

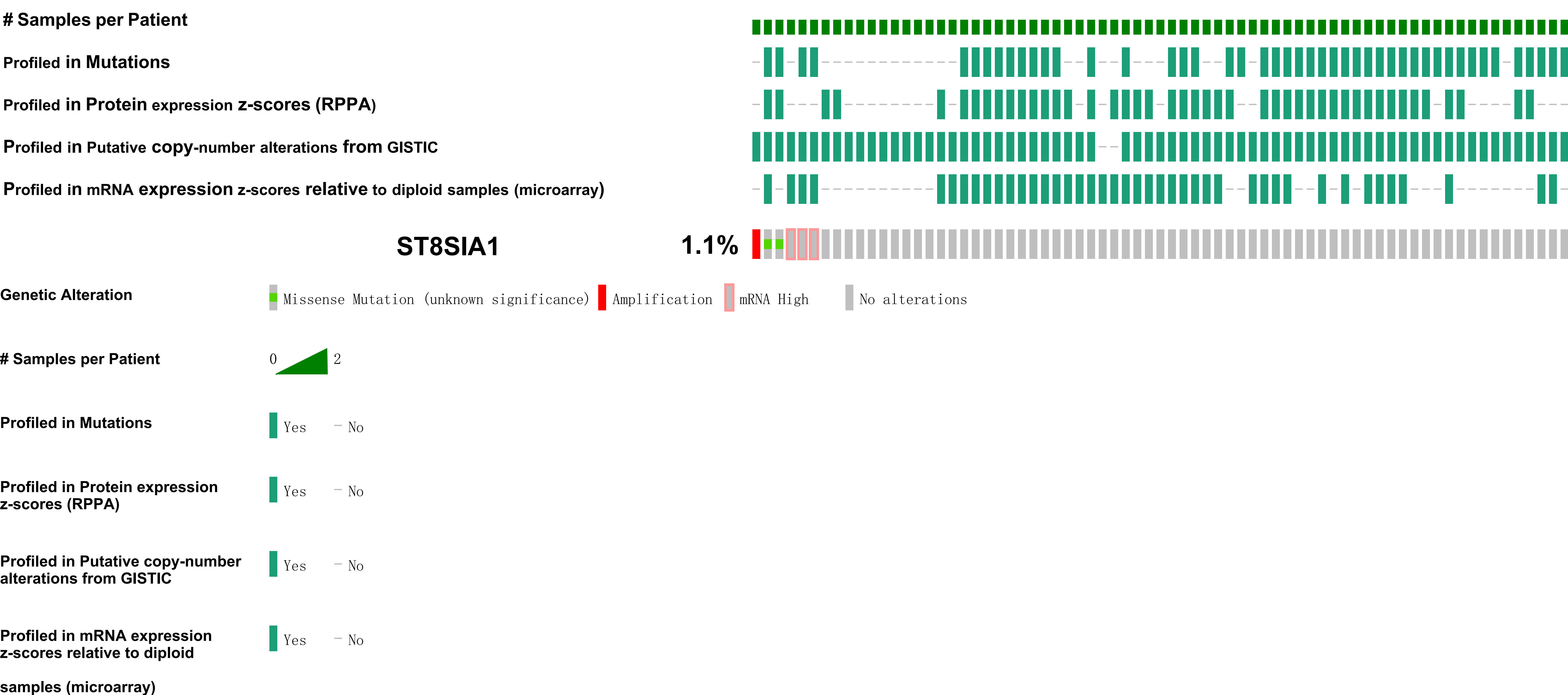

Samples per Patient
Profiled in Mutations
Profiled in Protein expression z-scores (RPPA)
Profiled in Putative copy-number alterations from GISTIC
Profiled in mRNA expression z-scores relative to diploid samples (microarray)
ST8SIA1
1.1%
Genetic Alteration
Missense Mutation (unknown significance)
Amplification
mRNA High
No alterations
Samples per Patient
0
2
Profiled in Mutations
Yes
No
Profiled in Protein expression z-scores (RPPA)
Yes
No
Profiled in Putative copy-number alterations from GISTIC
Yes
No
Profiled in mRNA expression z-scores relative to diploid samples (microarray)
Yes
No
